# Prasinovirus Attack of Ostreococcus Is Furtive by Day but Savage by Night

**DOI:** 10.1128/JVI.01703-17

**Published:** 2018-01-30

**Authors:** Evelyne Derelle, Sheree Yau, Hervé Moreau, Nigel H. Grimsley

**Affiliations:** a Integrative Marine Biology Laboratory (BIOM), CNRS UMR7232, Sorbonne Universities, Banyuls-sur-Mer, France; University of California, Irvine

**Keywords:** Phycodnaviridae, NCLDV, prasinophytes, Mamiellophyceae, Ostreococcus tauri virus 5, microbiology, marine microbiology

## Abstract

Prasinoviruses are large DNA viruses that infect diverse genera of green microalgae worldwide in aquatic ecosystems, but molecular knowledge of their life cycles is lacking. Several complete genomes of both these viruses and their marine algal hosts are now available and have been used to show the pervasive presence of these species in microbial metagenomes. We have analyzed the life cycle of Ostreococcus tauri virus 5 (OtV5), a lytic virus, using transcriptome sequencing (RNA-Seq) from 12 time points of healthy or infected Ostreococcus tauri cells over a day/night cycle in culture. In the day, viral gene transcription remained low while host nitrogen metabolism gene transcription was initially strongly repressed for two successive time points before being induced for 8 h, but during the night, viral transcription increased steeply while host nitrogen metabolism genes were repressed and many host functions that are normally reduced in the dark appeared to be compensated either by genes expressed from the virus or by increased expression of a subset of 4.4% of the host's genes. Some host cells underwent lysis progressively during the night, but a larger proportion were lysed the following morning. Our data suggest that the life cycles of algal viruses mirror the diurnal rhythms of their hosts.

**IMPORTANCE** Prasinoviruses are common in marine environments, and although several complete genomes of these viruses and their hosts have been characterized, little is known about their life cycles. Here we analyze in detail the transcriptional changes occurring over a 27-h-long experiment in a natural diurnal rhythm, in which the growth of host cells is to some extent synchronized, so that host DNA replication occurs late in the day or early in the night and cell division occurs during the night. Surprisingly, viral transcription remains quiescent over the daytime, when the most energy (from light) is available, but during the night viral transcription activates, accompanied by expression of a few host genes that are probably required by the virus. Although our experiment was accomplished in the lab, cyclical changes have been documented in host transcription in the ocean. Our observations may thus be relevant for eukaryotic phytoplankton in natural environments.

## INTRODUCTION

The Mamiellophyceae is a class of eukaryotic unicellular green algae whose phylogenetically diverse members have been particularly successful in colonizing the world's oceans (1, 2). Their tiny cell sizes (3), global dispersion, and ease of laboratory culture (4, 5) render them attractive as models for interdisciplinary studies in marine biology. In addition, the complete genomes of several species in the genera Ostreococcus, Bathycoccus, and Micromonas have been characterized (6), permitting their detection in metagenomic data collected in microbial fractions of environmental seawater fractions (1). Numerous species in this group of algae are infected by prasinoviruses (1, 7), large double-stranded DNA (dsDNA) viruses in the family Phycodnavirideae. While viruses infecting Micromonas spp. have been known for some time (8), those infecting Ostreococcus and Bathycoccus were discovered more recently (9–13). Several of these prasinoviral genomes have now been characterized, and they are typically about 200 kb long, carrying about 250 genes.

In aquatic environments in general, viruses play an important role in regulating the population of diverse phytoplankton and affect carbon and nutrient cycling by lysing susceptible host cells (14), but much remains to be discovered about their biology. In cyanobacteria, for example, diurnal regulation of host cell lysis has been observed (15–17). Phycodnaviruses are nucleocytoplasmic large DNA viruses (NCLDVs) that infect many species of eukaryotic algae. The best-characterized of these are Paramecium bursaria Chlorella virus 1 (PBCV-1), a species infecting freshwater Chlorella, which is also a symbiont of the ciliate Paramecium bursaria (18), and Emiliania huxleyi viruses (19), which infect the marine haptophyte unicellular alga Emiliania huxleyi, well known for its extensive oceanic blooms.

The life cycle of Ostreococcus tauri virus 5 (OtV5), with its typical icosahedral particle morphology, 8-hour-long latent period, and small burst size of 25, cultured with its host in continuous light, was first described by Derelle et al. (9). Numerous studies describing viral growth in related prasinoviruses infecting Micromonas have been made previously using a day-night cycle (20–27), and recently Demory et al. (28) revealed temperature to be a key factor in these interactions, but detailed molecular analyses were not the main objective of these studies. In the present study, we aimed to reexamine growth of OtV5 in a more-natural light regime (12 h light/12 h dark) and to characterize gene expression from the host and algal genomes by transcriptome sequencing (RNA-Seq) analyses.

## RESULTS

### Growth of host cells and virus after infection at different times.

**FIG 1:**
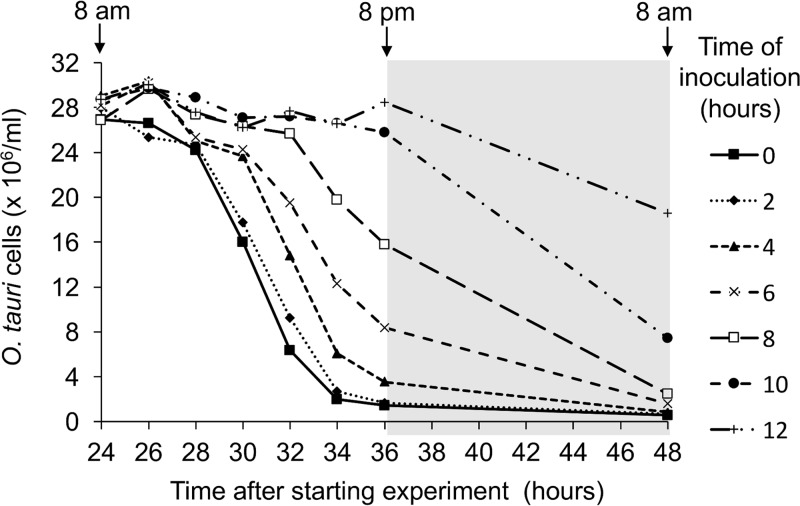
Time course of lysis of *O. tauri* cultures partially synchronized by a 12:12 light/dark cycle and inoculated with OtV5 (MOI, 5) at different times during the previous day, as indicated in the adjacent key. Note that almost complete lysis of cells occurred at 36 h postinoculation (hpi) only when cultures were inoculated on the previous day at 8 a.m. (time zero, filled squares with continuous line) or 10 a.m. (time 2, fine dotted line with filled diamonds).

A partial synchronization of *O. tauri* growth was previously reported under a 12:12 light/dark (L/D) cycle (29). In these cultures, cells were in G_1_ phase during most of the light phase and progressively entered in S phase and mitosis at the end of the day. The division of the population occurred during a period of 2 h before and 2 h after the light-to-dark transition. In such synchronized cultures, the effect of inoculating cultures at different times during the day was thus tested in a preliminary experiment, to find the best time to inoculate the cultures. For that purpose, *O. tauri* cultures were infected with OtV5 every 2 h during the light phase (Fig. 1). Cell lysis was almost complete at the end of the second day (36 h later) when the infection occurred in the first 4 h of the light period. In contrast, when infection occurred later, for example, after 10 to 12 h of light, no cell lysis was observed at 36 h after the start of the experiment and lysis was delayed until the next day.

### Virus-host infection dynamics.

**FIG 2:**
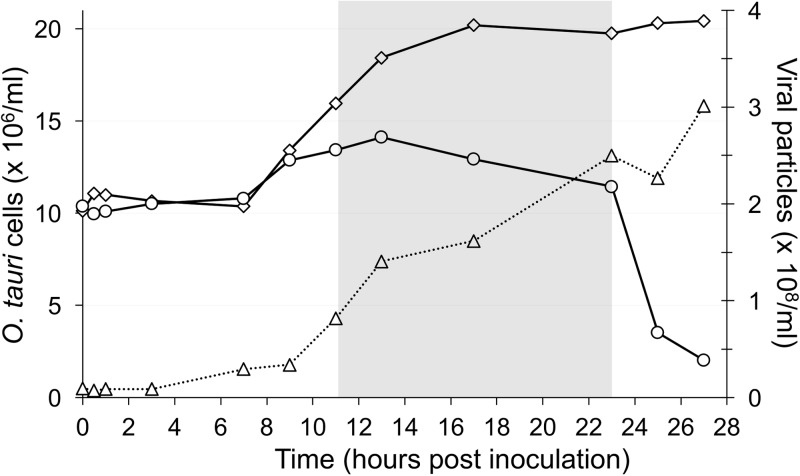
Growth curves of *O. tauri* cultures and OtV5. Open diamonds, uninfected *O. tauri*; open circles, *O. tauri* infected by OtV5; open triangles, OtV5 production.

**FIG 3:**
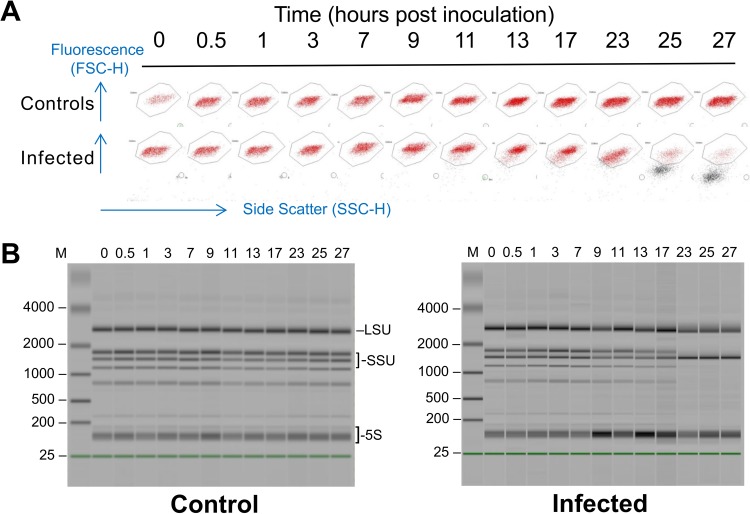
Cytograms and electropherograms of cells made during the time course of infection. (A) Visualization of *O. tauri* cells by flow cytometry in healthy (control) or infected (inoculated at 1 h after the start of the light period) samples of cultures taken at different times (hours postinoculation) during host and viral growth during the RNA-Seq analysis, shown as a composite of 24 excerpts from separate cytograms. Healthy *O. tauri* cells are seen as red fluorescent points clustering in the window shown, whereas lysing cells can be seen as dark points underneath the window with reduced fluorescence and side scatter that begin to appear in infected cultures at 9 hpi, becoming suddenly stronger at 25 hpi, and rising to a maximum at 27 hpi. Blue arrows show the directions of the relative values plotted in the *x* and *y* axes of the cytograms (side scatter gives an indication of cell size, and fluorescence represents the autofluorescence of chlorophyll). (B) Electropherograms of RNA extractions from healthy (control) or infected cultures on an Agilent 2100 Bioanalyzer, dark bands showing mainly abundant rRNAs. In infected cultures, the extracted RNA is partly degraded after 23 to 27 hpi, when most of the host cells are undergoing lysis. M, molecular weight marker track. Likely positions of abundant RNAs are shown on the right side of the left picture: LSU, unresolved bands of nuclear (nuc), chloroplastic (cp), and mitochondrial (mt) large ribosomal subunit RNAs (2821, 2858, 2585 bp); SSU, small ribosomal subunit RNAs (nuc-1738, cp-1569, mt-1460); 5S, unresolved bands of 5.8S, 5S rRNAs, and diverse tRNAs.

In order to have a complete viral lysis cycle within two working days, we infected cells using a multiplicity of infection (MOI) of 10 per cell 1 h after “dawn,” giving 11 h of light before the dark period. Under these conditions, infected cells did not start to undergo lysis at 8 h postinoculation (hpi) as observed previously under continuous light (9) but remained intact until after “nightfall.” Uninfected and infected cells started to divide at 7 hpi, but cell growth in infected cultures was strongly reduced after 9 hpi (Fig. 2). Control cells divided 7 to 17 hpi, doubling in cell density, but only about 40% of infected cells divided, reaching a maximum at 13 hpi and then declining slowly until 1 h after “dawn,” when about 80% of the remaining cells were lysed in the period of 23 to 25 hpi (Fig. 3). By 27 hpi, the “infected” population was reduced to about 7% of the control.

In inoculated cultures, given that the MOI was 10, we observed that most viruses adsorbed to each cell immediately after inoculation, since the density of particles measured by flow cytometry appears to drop to 1/10 of that in the inoculum. Few viruses were released from host cells by 9 hpi, but many more were released in the period of 9 to 13 hpi, and the viral population continued to increase until the end of the experiment, when the total number of virus particles was 25 to 30 times higher than the number of host cells inoculated at 0 hpi, in good agreement with the burst size of 25 calculated previously (9).

### Differentially expressed (DE) host genes.

mRNAs of all samples were analyzed using RNA-Seq technology, and host and viral gene expressions of control and infected cells were compared at each time point. With the parameters used in the analysis (see Materials and Methods), only 323 host genes were significantly differentially regulated at any one time point using the chosen analytical parameters (Table 1; see Materials and Methods and Data Set S1 in the supplemental material).

**TABLE 1:** Numbers of differentially expressed *O. tauri* genes^*a*^

DE pattern	No. of genes DE			
	Total	Up	Down	Up and down
All DE patterns	323	230	63	30
DE only once	207	151	56	0
DE at least two nonconsecutive times	24	7	2	15
DE at least two consecutive times	92	72	5	15

a DE, differential expression or differentially expressed; up, all DE genes were upregulated; down, all DE genes were downregulated; up and down, regulation of DE genes varied across the time course.

**FIG 4:**
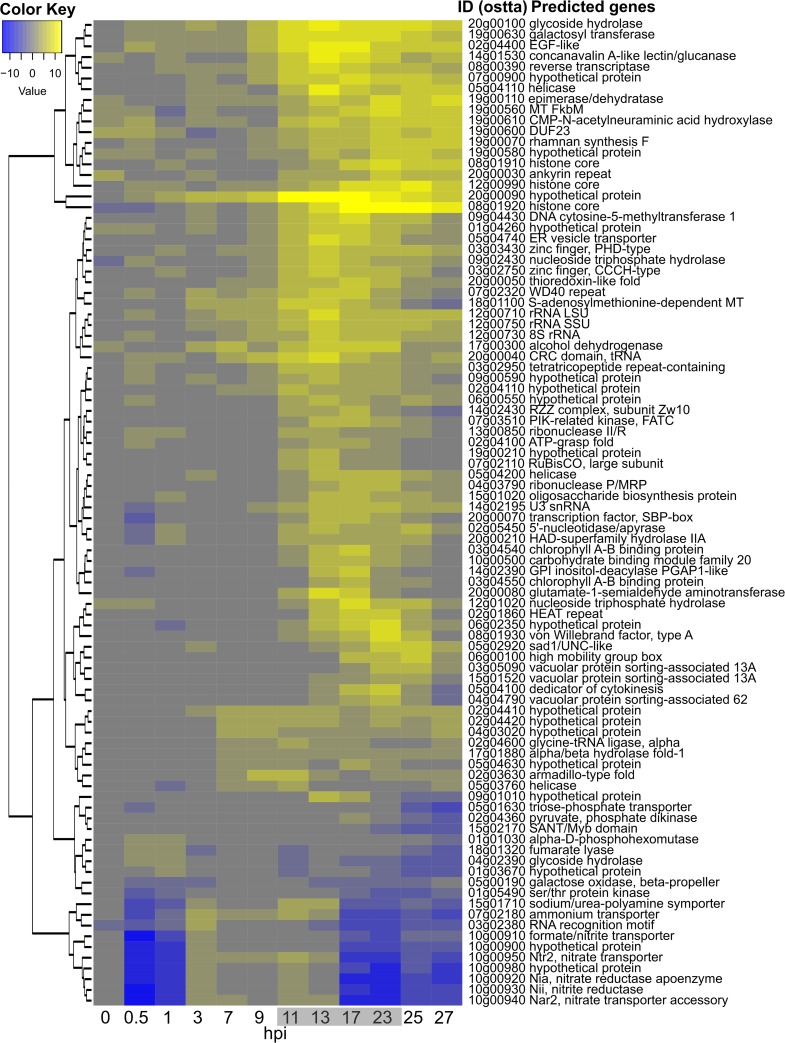
Differentially transcribed *O. tauri* genes during a 27-hour infection time course. Time (in hours postinfection) is shown along the abscissa, with time points sampled in the dark shown with a gray background, and rows represent DE genes clustered according to log_2_-fold changes in expression (a color key is shown in the upper left; see Data Set S2 in the supplemental material for a detailed list of genes).

**FIG 5:**
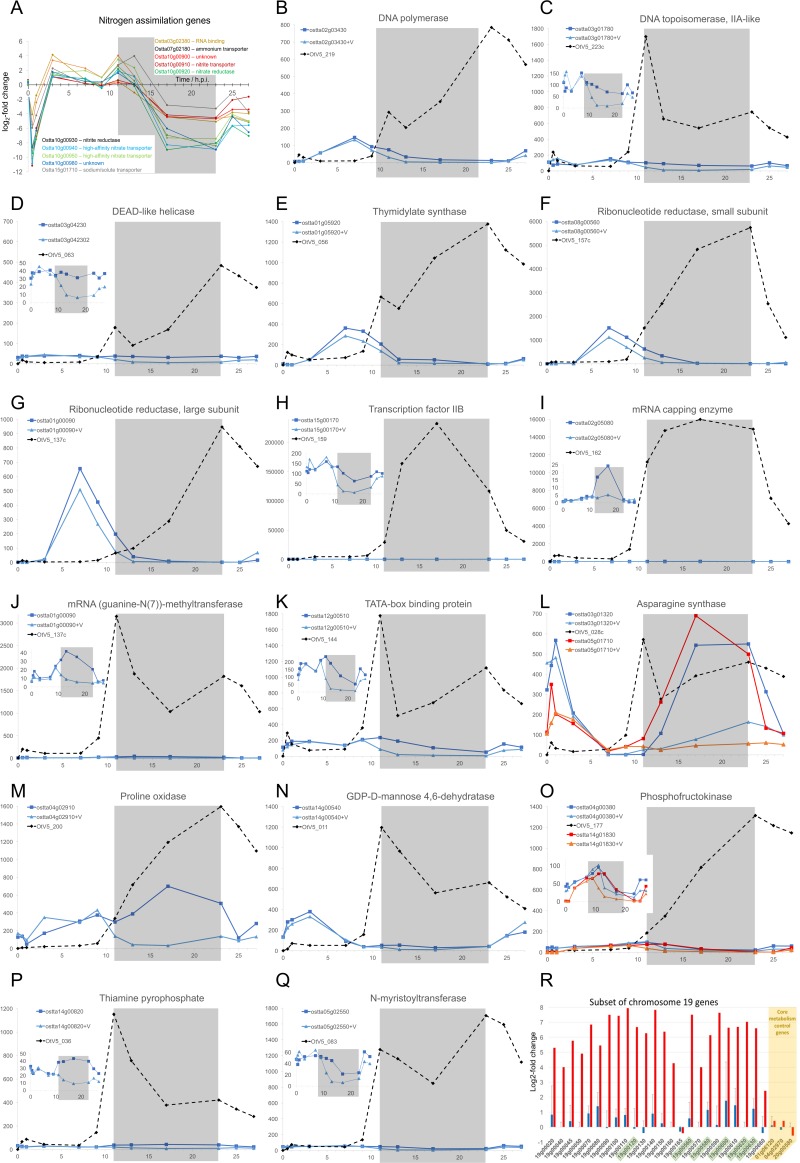
Expression of healthy, infected *O. tauri* and OtV5 genes. Inoculation with the virus (0 hpi) was done 1 h after daytime (light) started. The gray shaded area indicates the night (dark) period. Genes are labeled by their locus identifiers from public databases. (A) Differential expression (ordinate, log_2_-fold change; abscissa, time in hours postinfection [hpi]) of host genes regulated coordinately with those in the nitrogen assimilation gene cluster. (B to Q) Comparisons of genes sharing a similar putative function expressed from healthy host cells (square data points, darker colors), infected host cells (triangular data points, lighter colors), or OtV5 (diamonds, dashed lines). Gene identifiers are shown in insets with the curve colors and symbols, curves from virus-inoculated cultures being additionally distinguished by “+V.” Ordinates show the normalized levels of transcript abundance (in fragments per kilobase of exon per million fragments mapped [FPKM]). When viral transcripts were much more abundant than host transcripts, the profiles of host gene expression are shown as insets at appropriate scales. (R) Comparisons of gene expression (log_2_-fold changes) between earlier (0 to 3 hpi, blue bars, lines show standard deviations [SD] for 4 time points) or very late (25 to 27 hpi, red bars, average of 2 time points) stages of infection for chromosome 19 genes previously identified as being overexpressed in resistant lines (84). The expression of 3 control housekeeping genes is shown on the right in a yellow background (ostta01g06120, DNA-directed RNA polymerase, beta-subunit; ostta04g02970, ribosomal protein L1; ostta20g00580, E3 ubiquitin ligase, SCF complex). The comparisons for each gene were made at corresponding times after “daybreak” on days 1 and 2. Only 6 (identifiers boxed in green) of the 24 chromosome 19 genes were classed as differentially expressed using the chosen criteria for DE in the current work.

Given the high number of sampled time points in this experiment (24 mRNA libraries were sequenced), no replicates were done. To palliate this, only genes whose expression was differentially regulated at two or more consecutive sampling times were retained. Application of this criterion decreased to 92 the number of host genes that were considered to be regulated (Table 1 and Fig. 4). Most of them (72 genes) were only upregulated whereas 5 were only downregulated. Fifteen other genes were also regulated in the opposite direction at least once in the course of the experiment, albeit 13 having consecutive regulations at two successive times in the same orientation (up or down) (Table 1). Most of the regulated genes were individually dispersed in the genome except for a cluster of 7 genes, including the nitrate/nitrite transporter/reductase cluster previously described (30) and a group of genes on chromosome 19 (see below). Among the 92 genes, 77 (80%) had a potential identified function (see Data Set S2 in the supplemental material), but no clear metabolic pathways could be identified except for the nitrate/nitrite cluster mentioned above and present on chromosome 10 (Fig. 4 and 5A). This tandem organization indicated a possible selective pressure for optimization of nitrate uptake and assimilation by *O. tauri*, although experimental evidence for such a coordinated expression of these genes is currently lacking. Interestingly, in our experiment their expression was first strongly inhibited during at least the first hour postinfection and then upregulated to the same level as that of the control until 17 hpi and, finally, again strongly inhibited (Fig. 4 and 5A).

rRNA gene transcripts were much more abundant in infected cells than in healthy cells during the infection process (Fig. 4).

### Early fluctuations in host transcript abundance.

Disregarding the above requirement for DE in the same direction at two consecutive points, we observed that a small number of host genes showed up-downregulation or down-upregulation at two early time points after infection. Eight genes showing DE at one time point were upregulated at 0 to 1 hpi and then downregulated (up-down), and 7 genes showed downregulation at 0 to 1 hpi and then upregulation (data not shown). Most of these proteins are predicted to have regulatory functions (4 transporters, 3 nucleic acid binding proteins, 2 transcription factors, 2 unknowns, 1 kinase, 1 ATPase).

### Expression of viral genes.

**FIG 6:**
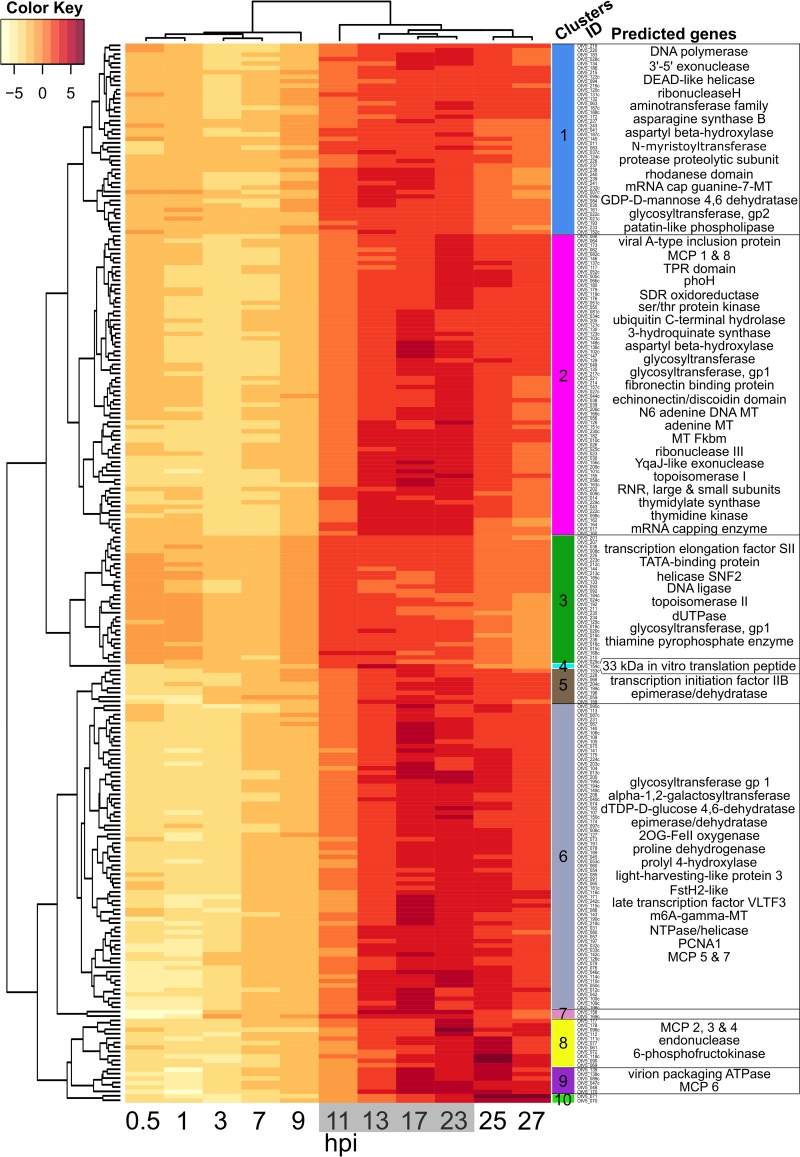
Timing of OtV5 relative gene transcription during infection. Time (in hours postinfection) is shown along the abscissa, with time points sampled in the dark shown with a gray background. Overall viral gene expression increased strongly after 11 hpi. Rows represent OtV5 genes clustered according to the variation in their relative expression over time (to the left of the dendrogram) and by the relative expression pattern of each sample (above the dendrogram). The “clusters ID” column shows the identity of each gene, providing a link to Data Set S3 in the supplemental material for a detailed list of genes. Only genes with a predicted function (33% of OtV5 coding sequence [CDS], so lines do not correspond with the 247 CDS) are listed in the right-hand column, to provide information about the kinds of functions that appear in that cluster. The color key shows regularized log (rlog)-transformed gene fragment counts centered to the mean of each gene (row means). See Materials and Methods and Data Set S3 for a detailed list of genes.

All of the viral genes were expressed during the life cycle, except for most of those present in the long terminal inverted repeats (TIRs). Clustering the data (Fig. 6) revealed successive functional groups of genes. The expression pattern of viral genes in infected cultures occurred in two phases (Fig. 6): phase I, from 0 to 9 hpi with low viral transcription (<6% of reads mapped to OtV5), corresponding to the start of cell division before the light/dark transition; and phase II, occurring after 11 to 27 hpi with high viral transcription (up to 66% of reads mapped to OtV5). During the first phase in the light, two clusters of phase I genes (clusters 3 and 1) stood out as more strongly expressed than the others. Cluster 3 was the strongest, concerning genes involved mainly in controlling transcription initiation and nucleotide processing, whereas cluster 1 contained a mixture of functions. Both of these clusters contained genes involved in DNA replication. The majority of phase II viral gene expression can be seen to occur probably when host DNA replication has been completed (29) at 13 to 23 hpi, in clusters 2 and 6, whereas clusters 8, 9, and 10 contain genes that are highly expressed very late in infection. Phase II also contains genes classically involved in late virus particle development, such as major capsid protein-like (MCP), viral A inclusion body protein, and virion packaging ATPase. At the end of phase II, all viral genes were expressed, except for 3 of the 4 genes in each TIR (OtV001c, OtV002c, OtV003c, OtV004, OtV245, OtV246, and OtV247 were not expressed).

The most expressed viral gene was annotated as encoding a 33-kDa *in vitro* peptide translation (see Data Set S3 in the supplemental material) whose function is not clear but which was also reported to be massively expressed in the closely related chlorella viruses (31). This gene was highly expressed throughout the infection. Among the 8 MCP-like genes, copies 1 to 7 began to be expressed late in the second phase, between 23 and 25 hpi, whereas copy 8 was expressed earlier (Fig. 6; see also Data Set S3 in the supplemental material).

Several genes with similar predicted functions are present in both the virus and the host genomes. The regulation of their respective expressions showed two patterns. The first pattern showed the highest expression of the host gene during the light phase and the highest viral gene expression in the dark, when the host gene counterpart expression was low. The virus thus appeared to be autonomous for some of the functions necessary for its growth during the night (Fig. 5B to Q). For example, this was observed for the two subunits of the ribonucleotide reductase and for the DNA polymerase (Fig. 5F and G). In the second pattern, the expression of the host genes was inhibited in the infected cells compared to the control, whereas the virus genes were expressed (Fig. 5D, C, I, J, L, O, and P), again appearing as a compensation of the host gene inhibition.

## DISCUSSION

Several previous detailed reports on the life cycles of large DNA viruses infecting green microalgae have been done in continuous illumination (9, 32–36), which promotes rapid growth of the host and virus, but since all of these algae have evolved in a diurnal cycle, we decided to perform this study in a 12-h light and 12-h dark “day” and “night” cycle. In healthy cells, under these conditions, the general pattern of gene transcription is quite different in the daytime, when photosynthesis is in progress, and at night, when stored energy is being used, this rhythm being observed both in the laboratory (37) and in the environment (38).

Whereas under continuous illumination there was a burst of viruses released at 8 hpi (9), in the light-dark cycle, the timing of the host cell lysis was variable, with some cells lysing during the night but most of the cells dying after illumination of the cells the following morning (Fig. 2). Under these conditions, it does not really make sense to think of the “burst” time as a fixed period. It probably also varies according to the temperature and, in nature, according to the seasons in temperate latitudes. Several authors have investigated the effects of host cell cycle (39) or different environmental variables on viral life cycles (20, 40–43), but appropriate tools were not available or not used for molecular analyses in these species. Using gene-specific probes or biochemical analyses, E. huxleyi viruses were shown to affect certain host metabolic pathways (44, 45), but diurnal variations were not discernible in this system. Our observations agree well with those of Brown et al. (22) on the related prasinovirus Micromonas virus MpV-Sp1; these authors observed a peak of viral production about 24 h after infection. Furthermore, they showed that host cell lysis was delayed in prolonged dark periods and confirmed their observations on host cell densities and virus production using molecular probes.

In natural populations of phytoplankton as in culture, cell growth responds strongly to light/dark periodicity (38, 46). Our data support the notion that viral gene transcription is rather quiescent during the day and increases rapidly at the onset of the dark when host DNA replication is being completed, thereafter remaining strongly expressed. Many of the genes that were significantly expressed in the quiescent phase are abundant in the active later phases, suggesting that their quiescent expression may reflect to some extent leaky general suppression levels. However, the heatmap clustering revealed that viral genes for nucleic acid processing and transcription do appear to be more abundant than other messages in the first phase (Fig. 6, clusters 1 and 3), although these genes continue to be expressed among the late genes. For example, transcription factor IIB (TFIIB), a conserved gene in eukaryotes and many large DNA viruses that is part of the core transcriptional machinery (47), was very highly expressed at night (Fig. 5H). At night, viral genes probably essential for the late stages of viral growth appeared to compensate for gene functions that were normally turned down at night, including functions probably important for DNA replication and amino acid metabolism, while transcripts likely encoding virion assembly and glycosylation were highest in the latest time points (Fig. 6). Although only arginine synthase and proline oxidase showed significantly different levels between control and infected cells at one time point (see Data Set S1 in the supplemental material), insufficient for our requirement of consecutive times, the strength of the coordinate swings in expression shown in Fig. 5 for numerous genes clearly intimates that the viral metabolism predominates, justifying our approach of numerous sampling times. Some host amino acid synthesis genes normally expressed in the dark were turned down in the dark in virus-infected cultures, but their viral counterparts were then upregulated (Fig. 5L and M). Viral proline oxidase (Fig. 5M) was probably acquired from its host genome (9), is known to produce ATP during stress responses in eukaryotes (48, 49), and is a possible source of energy for the virus. Phosphofructokinase is a key enzyme controlling the production of energy through glycolysis (50), and viral transcript levels in the dark rose to over 1 order of magnitude higher than those of the host (Fig. 5O).

### rRNA overexpression.

Although our extraction procedure was designed to isolate polyadenylated mRNA, some rRNA genes, which are always abundant in RNA extractions of active cells, were represented in our data. There is increasing evidence that rRNA transcripts can be polyadenylated in eukaryotes, including algae in the same phylogenetic order as Ostreococcus, such as Micromonas (51). rRNAs were overrepresented late in infection in *O. tauri*, compared with the control. At least three explanations are possible for this. First, it may result from the fact that the ribosome is a large and relatively stable subcellular structure that might persist better than the other cytoplasmic RNAs during the late viral infection, thereby preferentially protecting rRNAs that lie buried within it. Much of the available cellular RNA pool is likely to be used by the viral ribonucleoside-diphosphate reductase, an enzyme with two subunits that all prasinoviruses encode, to permit synthesis of prasinovirus DNA. This viral enzyme continued to be highly expressed during the night (Fig. 5F and G), when the equivalent host genes were shut down. Second, an overexpression of rRNA may be induced by the virus. U3, an RNA probably transcribed by RNA polymerase(pol) III (52) and essential for the first step of pre-rRNA processing (53), is apparently overexpressed during late viral infection. *O. tauri* RNA pol III is normally constitutively expressed, as it is required for many basic cellular functions (54), and indeed there is no significant difference observed in the expression of its controlling repressor, *ostta05g03220*, that encodes the orthologue of Maf1 (55). The apparent abundance of U3 suggests that it may not be dislodged from the rRNA for processing. The proteins UTP14 and DHR1 are required to dislodge U3 (56), but in *O. tauri* the putative orthologues of these genes (*ostta04g00770* and *ostta05g03760*, respectively) are not induced. Only 3 of the 161 annotated *O. tauri* ribosomal proteins were modestly overexpressed at one time point, and one other was modestly underexpressed (see Data Set S4 in the supplemental material). This would thus result in overproduction of unprocessed host rRNA precursors, potentially providing OtV5 with a rich source of nucleic acids by their degradation. Third, in yeast, where the dynamic, energetically demanding and complex process of ribosome biogenesis has been studied in detail (57), nutrient starvation or stress is known to shut down the synthesis of ribosomes via the conserved global regulatory target of rapamycin (TOR) pathway at the stage of initiation of transcription or pre-rRNA (57, 58). However, in our system rRNA appears to accumulate, and its processing occurs in an apparently normal way up to 23 hpi (Fig. 3). Recently Kos-Braun et al. (59) demonstrated an alternative pathway for blockage of rRNA processing at a later stage during the diauxic growth phase in yeast. When glucose is no longer available, casein kinase 2 (CK2, an orthologue of ostta12g02550 in *O. tauri* [60]) can phosphorylate TOR1, and partly processed rRNA products can accumulate in a resting (G_1_ or G_0_) stage. While this type of control also leads to accumulation of rRNA, Kos-Braun et al. show that the 5S moiety in yeast remains attached to the large rRNA subunit precursor, whereas in *O. tauri* the accumulated rRNAs look normal.

While our data favor the second hypothesis, further work is required to study this process in more detail, since it may be a pivotal switch governing the acquisition of sufficient cellular metabolites to re-source the biosynthesis of large viral genomes before the host cell bursts. A least two of the control steps of host rRNA production might occur by protein phosphorylation (phosphorylation of TOR by its controlling proteins either at the stage of pre-RNA initiation or at a later stage [59]) and were out of the scope of the current study. More-precise analysis of processing at the 5′ part of the pre-rRNA (the position of U3 binding) would also be desirable.

### Nitrogen assimilation.

The uptake and conversion of nitrate to its reduced form required for synthesis of amino acids constitute a complex and energetically demanding process (61). The expression of genes involved in the assimilation of nitrate, the only source of nitrogen in our culture medium, and many others of the N assimilation pathway are strongly differentially expressed throughout the course of infection, being first repressed and then induced and finally repressed (Fig. 4). These include numerous genes clustered together on chromosome 10 and a few genes scattered on other chromosomes (Fig. 5A). This is striking, because it is not related to the nitrogen sources available in the medium. In addition, none of the 3 cyclin-dependent protein kinase genes shown to be involved in N assimilation responses (62) showed differential expression, suggesting that this response is not functioning. There is an adequate level of nitrate in the culture medium used (no ammonium provided in L1; see Materials and Methods), so nitrate uptake and nitrate reductase genes should be highly active, as they are in the control. There are several possible nonmutually exclusive reasons for this repression, which might either be initiated as a host defense response or be the result of virally encoded products influencing N assimilation by this pathway.

Reduction of nitrate via nitrate reductase also leads to production of nitric oxide (NO) (63, 64), signaling reactive oxygen species (ROS) active in diverse species (65, 66), including algae (61), that is known to heighten the cellular defense responses of cells to stress (66–68). It is required for resistance to viruses of Arabidopsis (69) and rice (70), and ROS are also known to modulate the response of E. huxleyi to viruses (45). Since the nitrogen and carbon/phosphorus ratio for small green algal structural and metabolic requirements far exceeds that of the nucleic acid-rich large DNA viruses (71) and the cell is doomed to lysis, it may be advantageous for the virus to divert the resources usually used for protein synthesis toward nucleic acid synthesis, at the same time lowering the chance of detection by NO signaling that would initiate host defenses. If the TOR complex is targeted by the virus as suggested above and as shown recently in other host-pathogen systems (72, 73), this might also lead to TOR-controlled repression of the nitrogen assimilation genes (74). The coordinated regulation that we observed suggests the involvement of a global regulator, with opposing forces governing this control, provoking a strongly fluctuating response. However, the recent demonstration that certain prasinoviruses have acquired host genes that permit uptake of reduced nitrogen (75) suggests that this resource may also be limiting during infection and favors the notion that suppression of NO signaling is the reason for decreasing the uptake of nitrate.

### Is the replicative form of OtV5 chromatinized?

In several other host-virus systems, chromatinization of viral DNA that enters the nucleus is known to occur rapidly once the viral DNA enters the nucleus (76–78). The replicative form of OtV5 has not yet been investigated, but it very likely has a nuclear phase during its infection cycle, as OtV5 lacks a DNA-dependent RNA polymerase to transcribe viral genes (9). Herpes simplex virus (HSV), for example, is a dsDNA virus that probably replicates in the nucleus and is packaged in capsids as a linear molecule in the cytoplasm. During the HSV lytic cycle, the viral genome circularizes and nucleosomes form along its genome (79) in a highly dynamic way that is modulated by a viral transcription factor (80). The strong induction of all host histone core genes observed throughout the OtV5 life cycle strongly suggests that the OtV5 genome is chromatinized during replication of the viral genome and that viral replication continues throughout the dark cycle, when many photosynthesis-dependent host processes are shut down (81). Host S-adenosylmethyltransferase, an enzyme required for the majority of processes that modify DNA, RNA, histones, and other proteins, including those affecting replication, transcription and translation, mismatch repair, chromatin modeling, epigenetic modifications, and imprinting (82), was overexpressed in a similar way, suggesting that any of these pathways might be induced during viral infection. Its continued expression, also during the night, is likely necessary for the numerous pathways required for virus production.

### Induction of reverse transcriptase.

The *O. tauri* reverse transcriptase gene *ostta08g00390* was strongly induced (over 4 consecutive time points, and up to 420-fold at 13 h postinoculation) (Fig. 4; see Data Set S2 in the supplemental material) during the period when cell division is expected to occur (at the end of the day, from 2 h before dark and then for the following 6 h). This gene is predicted to encode the replicase/integrase of a putatively complete type I transposon (30, 83, 84) that is not usually active in healthy *O. tauri* cells. At 7 to 13 hpi, we observed a strong increase in the transcription of this gene. We hypothesize that the increase in transcription of this reverse transcriptase may be activated by the cellular stress response caused by the OtV5 attack, which may in turn activate transposition itself and the repeat retrotransposon in miniature (TRIM) on chromosome 19, leading to chromosomal rearrangements and possibly to activation of certain genes on chromosome 19 whose expression continues late in infection in those cells that subsequently become resistant to viral attack. Yau et al. (84) observed rearrangements on chromosome 19 and overexpression of genes on this chromosome in cell lines that had become resistant to OtV5 infection, and the karyotypes of these strains also suggest possible rearrangements and/or translocations on chromosome 19. This may additionally explain the presence of DNA in very large amounts in the pulsed-field gel electrophoresis (PFGE) gel, since long reads of that chromosome by reverse transcriptase from transposon long terminal repeats (LTR) might generate DNA intermediates that would not enter the gel (85–87). Recently, Blanc-Mathieu et al. (88) revealed the astonishing variability in the structure of chromosome 19 in natural populations of *O. tauri*. Whether or not rearrangements of this chromosome contribute to the acquisition of viral resistance is not yet clear and will be a subject for future investigations.

### Host genes induced very late.

While most of the host and viral differentially expressed genes showed increased transcription just after the beginning of the night time (Fig. 5 and 6), a time when we expect host DNA replication to be under way, surprisingly, a few host genes showed a second period of induction very late in infection, during the second half of the night and the morning of the next day, 17 to 27 hpi. Since several of them were also observed to be induced in OtV5-resistant lines of *O. tauri* (84), we compared the host genes identified in both experiments as being differentially expressed. Twenty-six genes were found to be differentially expressed at some stage in both of the analyses, and the expression of 11 of them was strongly upregulated in the last 13 to 27 hpi of the experiment. Since the majority of these genes (6/11) were located on the viral immunity chromosome first described by Yau et al. (84), we hypothesize that this expression originates from a subpopulation of resistant cells that have differentiated from the bulk of the susceptible cells, the latter being condemned to lysis and the release of viral progeny.

In summary, we have shown that in a natural light regime the life cycle of prasinoviruses in Ostreococcus in culture is biphasic, remaining quiescent by day but reaching full-scale activity at night, when new virus particles arise steadily at first and then rather suddenly in the morning. During the night, 239/247 (96.8%) of predicted viral genes are transcribed, and 323/7749 (4.2%) host genes are differentially expressed at some stage, the great majority (71%) being upregulated, in response to the viral attack. However, the pattern of host gene expression in the final phase of infection already suggests that a small population of host cells were adapting to become founders for resistance to OtV5. Detailed knowledge of host-virus interactions will be necessary for advancing our understanding of the everlasting war between hosts and their viruses in aquatic environments.

## MATERIALS AND METHODS

### Culture conditions and growth measurements.

The host strain Ostreococcus tauri RCC4221 (30, 83, 89) and the prasinovirus OtV5 (9) were used in all experiments. Cultures were grown in L1 medium (Bigelow Laboratory, NCMA, USA) diluted in 0.22-μm-filtered seawater under a 12/12 light/dark cycle (100 μmol photon/m^2^ s^−1^). Cell and viral counts were performed on a FACScan flow cytometer (Becton Dickinson, San Jose, CA, USA). *O. tauri* cells were counted according to their right-angle scatter and their red fluorescence emission due to the chlorophyll A pigment (90). OtV5 counts were determined by their right-angle scatter and their fluorescence after SYBR green I staining (91). For preparation of large quantities of viruses, 5 liters of an *O. tauri* exponentially growing culture (approximately 5 × 10^7^ cells ml^−1^) was inoculated with an OtV5 lysate. Lysed cultures were centrifuged at 8,000 × *g* for 20 min at 20°C and then passed through 0.22-μm filters to remove large cellular debris. Virus filtrates were concentrated by ultrafiltration with a 50,000 molecular weight (50K MW) size cutoff unit (Vivaspin 15 Turbo; Sartorius) to a final volume of 5 ml. The concentration of infectious particles was determined by a serial dilution assay.

To test for the effect of viral infection at different times during the day, *O. tauri* cultures in exponential growth phase were infected with purified OtV5 at an MOI of 5, and cell counts were determined over 48 h.

To perform the differential expression analysis, an *O. tauri* culture was acclimatized such that cell density doubled every day, from 10^7^ to 2 × 10^7^ cells/ml by flow cytometer counting, and by diluting the culture daily for 10 days. After this period of acclimation to maintain cultures in this rhythm of growth, 1.5 liters of *O. tauri* culture was prepared, and the cells were counted by flow cytometry and adjusted to a cell concentration of 10^7^ cells ml^−1^ by addition of L1 medium, and one half of the culture was infected 1 h after the beginning of the light phase with OtV5 at an MOI of 10. The cultures were then split into control and infected cultures, comprising 12 100-ml flasks for each condition. At 12 different times between 0 and 27 hpi, control and infected flasks were sampled to measure cell and viral densities by flow cytometry, and cells were harvested for RNA extraction (Fig. 2 and 3).

### RNA extraction and sequencing.

For RNA extraction, 50 ml of cells was harvested by centrifugation at 8,000 × *g* for 20 min at 20°C. The pellets were then flash frozen in liquid nitrogen and stored at −80°C. Total RNA was extracted using the Direct-zol RNA kit (Zymo Research) and checked for quality (Data Set S1B). Selection for polyadenylated RNA, library preparation, and sequencing were performed commercially (GATC Biotech AG, Germany). RNA libraries were sequenced on the Illumina Hi-Seq 2000 platform by multiplexing all samples on a single flow cell lane, which generated paired-end reads of 101 bp in length. RNA sequence reads were checked for quality using FastQC.

### Differential gene transcription analysis.

Transcriptome read pairs (fragments) were aligned using TopHat2 (92) (alignment parameters: -i 17 -I 3500, -G) to the annotated genome sequence of O. tauri RCC4221 (83) and OtV5 (9). The counts of fragments aligning to each gene were determined using the htseq-count function of HTSeq (93) with parameters “-m intersection-nonempty.” Fragments per kilobase of exon per million reads mapped (FPKM) were calculated for visualization of the expression of individual genes of interest. Differential gene expression analysis and data visualizations were performed in the R statistical environment (https://www.r-project.org/). Differential host gene expression analyses were performed on fragment count tables using the R package DESeq (93) to detect genes involved in OtV5 infection. Host gene transcription from each sampling time point of control uninfected cells was compared to that of infected cells using the DESeq function accepting genes as significantly differentially transcribed with an adjusted *P* value of <0.1. Candidate host genes involved in viral infection were accepted if >100 reads were assigned to the gene and if they were differentially transcribed in at least two consecutive time points. Heatmaps and the accompanying hierarchical clustering of O. tauri and OtV5 gene transcription were produced using the heatmap.2 function from the gplots R package. For O. tauri, we took as the heatmap input the log_2_-fold change values of DE genes outputted from the DESeq analysis. For OtV5, we took the fragment counts of all genes, excluding the terminal inverted repeats and 0 hpi as transcription was zero or negligible, transformed to a regularized log_2_ (rlog) scale using the DESeq2 package rlog function, which estimates the fragment counts proportional to the expected counts for genes for each sample based on the dispersion mean over the entire data set, and centered to the mean for each gene (row means) as the heatmap input. We designated OtV5 gene clusters by cutting the resulting hierarchical clustering tree (Euclidean distance) of the rlog-transformed mean-centered fragment counts to a depth that corresponded visually to prominent groups that covaried in their relative expression over time in the heatmap (cuttree function h = 5).

### Accession number(s).

O. tauri RCC4221 chromosome sequences can be found under GenBank accession numbers CAID01000001.2 to CAID01000020.2 (83), and gene annotations are also available from the Online Resource for Community Annotation of Eukaryotes (ORCAE; http://bioinformatics.psb.ugent.be/orcae/) under Ostreococcus tauri V2. The updated genome sequence and annotation of OtV5 are available under GenBank accession number EU304328.2. Transcriptomic data used in this study are available under BioProject accession number PRJNA400530.

## Supplementary Material

Supplemental material
