# Supplementary material for: Prasinovirus Attack of Ostreococcus Is Furtive by Day but Savage by Night
Source: J Virol. 2018 Jan 30;92(4):e01703-17. doi: 10.1128/JVI.01703-17 (PMC5790953; doi:10.1128/JVI.01703-17)
Supplement: Supplemental material [file supp_92_4_e01703-17__index.html]

Supplemental material 

# Prasinovirus Attack of Ostreococcus Is Furtive by Day but Savage by Night

## Supplemental material

- Supplemental file 1 -

  Data Set S1 (Differentially expressed host genes, comparing infected cells with uninfected cells.)

  XLSX, 33K
- Supplemental file 2 -

  Data Set S2 (Host genes expressed differentially at two or more successive time points.)

  XLSX, 86K
- Supplemental file 3 -

  Data Set S3 (OtV5 gene expression, using FPKM values clustered according to Euclidean distance, as shown in Fig. 6.)

  XLSX, 99K
- Supplemental file 4 -

  Data Set S4 (Transcript abundance values for RNA reads from the control and from cultures inoculated with OtV5 at time zero.)

  XLSX, 2.9M
